# Camouflaging bacteria by wrapping with cell membranes

**DOI:** 10.1038/s41467-019-11390-8

**Published:** 2019-08-06

**Authors:** Zhenping Cao, Shanshan Cheng, Xinyue Wang, Yan Pang, Jinyao Liu

**Affiliations:** 1 Institute of Molecular Medicine, State Key Laboratory of Oncogenes and Related Genes, Shanghai Institute of Cancer, Renji Hospital, School of Medicine, Shanghai Jiao Tong University, 200011 Shanghai, China; 2 Department of Ophthalmology, Shanghai Ninth People’s Hospital, School of Medicine, Shanghai Jiao Tong University, 200011 Shanghai, China; 3 Shanghai Key Laboratory of Gynecologic Oncology, Department of Obstetrics and Gynecology, Renji Hospital, School of Medicine, Shanghai Jiao Tong University, 200011 Shanghai, China

**Keywords:** Biomaterials - cells, Cell delivery, Cancer imaging, Applied microbiology, Biomedical engineering

## Abstract

Bacteria have been extensively utilized for bioimaging, diagnosis and therapy given their unique characteristics including genetic manipulation, rapid proliferation and disease site targeting specificity. However, clinical translation of bacteria for these applications has been largely restricted by their unavoidable side effects and low treatment efficacies. Engineered bacteria for biomedical applications ideally need to generate only a low inflammatory response, show slow elimination by macrophages, low accumulation in normal organs, and almost unchanged inherent bioactivities. Here we describe a set of stealth bacteria, cell membrane coated bacteria (CMCB), meeting these requirement. Our findings are supported by evaluation in multiple mice models and ultimately demonstrate the potential of CMCB to serve as efficient tumor imaging agents. Stealth bacteria wrapped up with cell membranes have the potential for a myriad of bacterial-mediated biomedical applications.

## Abstract (web summary)

The use of engineered bacteria for biomedical applications is limited by side effects such as inflammatory response. Here the authors engineer cell membrane coated bacteria as in vivo tumor imaging agents, and show that these generate a lower inflammatory response and reduced macrophage clearance.

## Introduction

Microbes are ubiquitous and are essential components of ecosystems, in particular playing critical roles in the maintenance of mammalian homeostasis and host health^1^. Microorganisms such as viruses and bacteria exhibit promising potential as therapeutics, bioimaging, and diagnostic agents because of their unique characteristics including genetic manipulation, rapid proliferation, and disease site targeting specificity^2–4^. Although viruses have been utilized for genetic therapeutics to permanently overexpress protein drugs or interfering RNAs in target cells^5,6^, the utility of viruses is largely limited by their rapid spread, difficulty to eliminate and potential to cause serious infections. Bacteria, another group of broadly used microorganisms, are relatively manipulable and safe, because they are easy to be modified genetically and can be controlled by antibiotics^7–9^. More importantly, various bacterial species are able to colonize specific body sites of mammalian hosts, for example the gut or the skin, as well as disease sites including abscesses and tumor tissues^8,10–14^. Based on these characteristics, bacteria have been widely administrated orally as probiotics to prevent pathogen colonization, or injected as bacterial-mediated tumor therapeutics^8,15–20^. More recently, a variety of bacterial therapeutics have been implemented in humans and entered into phase I clinical trials^7,21–23^. However, severe side effects may arise in a dose-dependent manner, despite the attenuated nature of the organisms used^24^. In addition, the therapeutic efficiency remains low, as most of the administrated bacteria are cleared by the reticuloendothelial system (RES) before colonizing specific target sites. As reported, phase I clinical trials of *Salmonella* VNP20009 were terminated owing to its low therapeutic efficacy and the emergence of high toxicity^21,23^. The current barriers for in vivo applications of bacteria are low treatment efficiency resulting from rapid clearance and unavoidable side effects caused by immunogenicity.

Surface decoration is an efficient method to enhance the in vivo performance of implanted biomedical devices and therapeutic agents. A variety of materials such as synthetic polymers, liposomes, and biological membranes have been explored for surface modification to improve biocompatibility, targeting ability, and blood circulation. As a gold standard coating material, polyethylene glycol has been extensively used to decrease protein nonspecific absorption and prolong blood circulation time^25^. Liposomes^4,26^ and natural cell membrane derived vesicles have been used to coat nanoparticles to improve biocompatibility and targeting ability^27–33^. For instance, erythrocyte membrane material slowed the clearance of poly(lactic-co-glycolic acid) nanoparticles significantly due to the presence of CD47 protein, a self-marker, on the membrane^34,35^. We therefore hypothesized that surface decoration of bacteria would be an alternative approach to design bacteria with an improved safety profile and enhanced efficiency for bacterial-mediated diagnosis, imaging, and therapy.

**Fig. 1:**
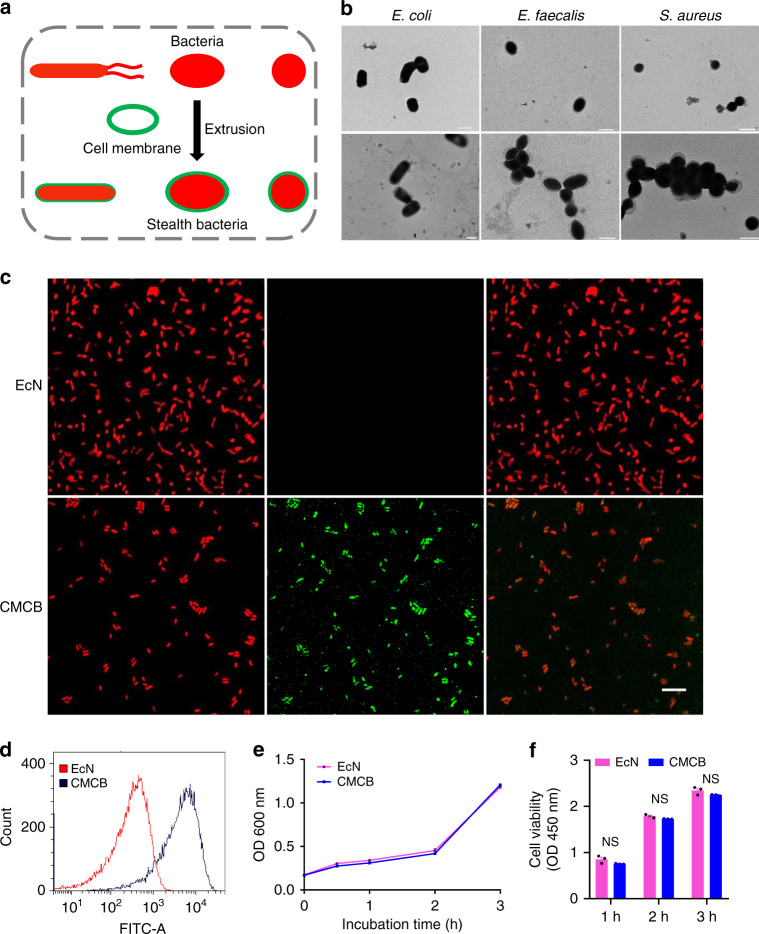
Preparation and characterization of CMCB. **a** Schematic illustration for the preparation of CMCB by extruding bacteria with cell membranes. **b** Representative TEM images of uncoated bacteria and CMCB. Scale bar, 1 μm. **c** LSCM images of EcN and CMCB. The red channel shows EcN producing mCherry, the green channel shows cell membranes conjugated with FITC-affinity anti-CD47-antibody, and the merge (orange) shows CMCB. Left mCherry, Middle FITC, Right merge. Scale bar, 10 μm. **d** Flow cytometric analysis of EcN and CMCB. **e** Growth curves of EcN and CMCB. Bacteria were cultured in LB medium at 37 °C and OD_600_ was measured at the indicated time points. **f** Bacterial viability analysis of EcN and CMCB by CCK-8 assays. Bacteria were incubated at 37 °C and viability was monitored by measuring OD_450_ at 1 h intervals. Error bars represent the standard deviation (*n* = 3). NS no significance

Herein, we report a strategy to generate stealth bacteria by camouflaging with cell membrane, which we call cell-membrane-coated bacteria (CMCB), prepared by simply extruding erythrocyte membranes with bacteria (Fig. 1a). We choose erythrocyte membranes considering the low immunogenicity and long circulating properties. This approach not only lowers the inflammatory reaction and side effects of CMCB as the bacterial immunogens are camouflaged, it also decreases the bodily clearance of CMCB because of the anti-phagocytic nature of erythrocyte membrane. Meanwhile, there are no significant effects on the bioactivities of the coated bacteria since the coating membrane is eventually removed during cell division of bacteria. Compared with uncoated bacteria, CMCB achieve (1) ~14 times higher blood reservation 48 h post-injection; (2) a lower inflammatory response; (3) lower accumulation in normal organs; (4) up to 42 times higher accumulation in tumor tissue; and (5) almost unaffected inherent bioactivities, as demonstrated in multiple mice models. This strategy is applicable to different strains such as gut microbes and therapeutic bacteria and suitable for coating with diverse cell membranes including erythrocyte, platelet, macrophage, neutrophil, and cancer cell membranes. We anticipate the camouflage of bacteria by wrapping with cell membranes to be a versatile approach for the preparation of biologically functional bacteria with improved safety and enhanced treatment efficacy and believe that the stealth bacteria coated with cell membranes represent a unique tool for biomedical applications.

## Results and discussion

### Preparation and characterization of CMCB

As a proof-of-concept study, we chose a well-known probiotic bacterium, *Escherichia coli* Nissle 1917 (EcN), a host bacterium which has been used for treating gastrointestinal disorders and solid tumors^15–18^. CMCB were prepared by simply fusing EcN with erythrocyte membranes via mechanical extrusion. Erythrocyte membranes were extracted from red blood cells of 6–8-weeks-old male Institute Cancer Research (ICR) mice following a previous method^36^ with minor modification (Supplementary Fig. 1). The resultant membranes were mixed with EcN and then extruded through a porous polycarbonate membrane (pore size: 1 μm). Transmission electron microscopy (TEM) images show a clear outer lipid shell on the surface of CMCB (Fig. 1b and Supplementary Fig. 2a, b). We speculated that the outer layer with thickness around 100 nm was attributed to the significant dehydration of the coated bacteria before TEM observation, which resulted in the collapse of the coating membranes. To verify this hypothesis, we used synthetic micro-particles (that cannot dehydrate) with a similar size to bacteria as controls. As shown in Supplementary Fig. 2c, d, the coating membranes show a thickness about 10 nm, which is in good agreement with the reported membrane width of erythrocytes. In contrast, uncoated EcN displayed a sharp edge with extended flagella. Similarly, bacteria with different shapes including elliptic *Enterococcus faecalis* (*E. faecalis*) and spherical *Staphylococcus aureus* (*S. aureus*) could be simply coated, showing the broad applicability of this approach to camouflage various strains (Fig. 1b and Supplementary Fig. 3). CMCB were further visualized by laser scanning confocal microscopy (LSCM) with the help of FITC-affinity anti-CD47-antibody labelling. The LSCM images shown in Fig. 1c demonstrate that EcN expressing mCherry (red) were quantitatively coated with cell membranes (green). Successful coating with cell membranes was also confirmed by flow cytometric analysis. As shown in Fig. 1d, the mean fluorescence intensity of CMCB after incubation with FITC-affinity anti-CD47-antibody was about 10 times higher than that of uncoated EcN, indicating that CMCB were coated with cell membranes. Additionally, western blotting shows the existence of self-marker of CD47 protein in CMCB, demonstrating the presence of coating membranes on the bacteria (Supplementary Fig. 4).

To examine whether the coating membranes have any effect on the viability of coated bacteria, growth curves of CMCB were recorded (Fig. 1e). Compared to uncoated EcN, CMCB grew at a similar rate and reached the same OD_600_ at 3 h. At the same time, bacterial viability was assessed using a cell counting kit-8 (CCK-8). Consistent with the growth curve results, there was no notable difference in viability between uncoated EcN and CMCB (Fig. 1f). Additionally, cells were stained with propidium iodide (PI) and imaged to evaluate the number of dead cells. LSCM imaging indicates that the bacteria were able to grow inside the membrane vesicles, demonstrating negligible effects on bacterial viability following coating with cell membranes (Supplementary Fig. 5).

### In vivo blood reservation

**Fig. 2:**
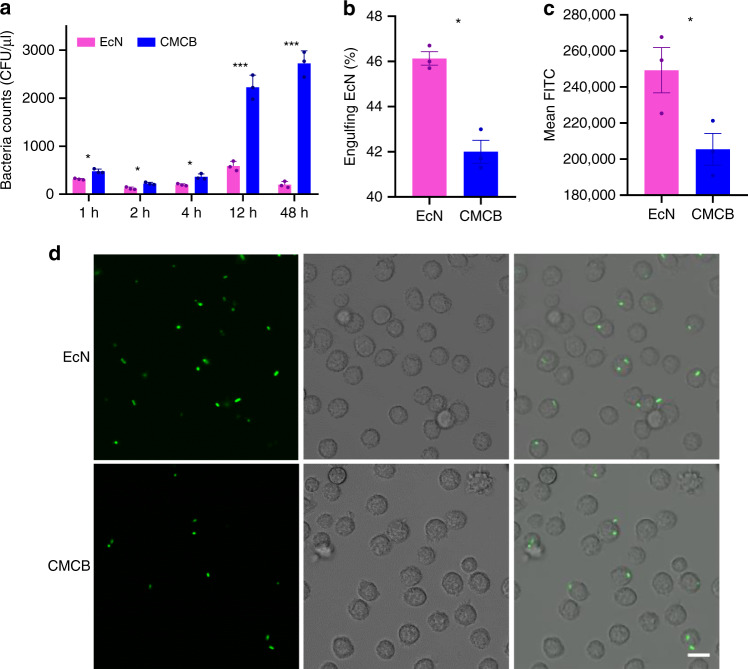
Evaluation of in vivo blood reservation. **a** In vivo blood reservation of bacteria. EcN or CMCB (1 × 10^7^ CFUs) were injected through the tail vein and blood was withdrawn intraorbitally at the indicated time points, diluted to 10^−2^ and spread onto LB agar plates. Plates were incubated at 37 °C for 24 h prior to enumeration. **b**–**d** Bacterial engulfment by primary peritoneal macrophages. Macrophages were co-cultured with EcN or CMCB at 37 °C with 5% CO_2_ for 1 h. **b** The percentage of macrophages containing bacteria and **c** the mean fluorescent intensity of the engulfed bacteria was measured by flow cytometric analysis. Error bars represent the standard deviation (*n* = 3). Significance was assessed using Student’s *t*-test, giving *p*-values, *p < 0.05, **p < 0.01, ***p < 0.005. **d** LSCM images of macrophages after co-incubated with EcN or CMCB expressing GFP at 37 °C with 5% CO_2_ for 1 h. Scale bar, 10 μm

We then investigated in vivo blood reservation of bacteria by injecting 1 × 10^7^ colony forming units (CFUs) of CMCB into the tail vein of ICR mice. Twenty microliters of blood from each mouse was collected intraorbitally at predetermined time intervals. Serial dilutions were prepared and then spread onto Luria Bertani (LB) agar plates, which were further incubated at 37 °C for 24 h before bacterial counting. Although the bacteria were eliminated rapidly followed injection, the amount of bacteria remained in blood from mice treated with CMCB was much higher than that of uncoated EcN. As shown in Fig. 2a and Supplementary Fig. 6, the CFUs from mice injected with CMCB at 12 and 48 h post-injection were around 4 and 14 times greater, respectively, than that of uncoated EcN, suggesting significantly increased retention of EcN in the blood circulation after wrapping with cell membranes.

The significantly improved blood reservation of bacteria by coating with cell membranes encouraged us to further explore the mechanism by which elimination was reduced. We asked the question whether the engulfment of EcN by the RES was inhibited after the bacteria were coated with cell membranes, as the RES has been recognized as the main clearance mechanism of intrusive microorganisms^37^. To facilitate the observations, EcN expressing green fluorescent protein (GFP) was constructed and cultured with primary peritoneal macrophages isolated from ICR mice^38^. The engulfment of bacteria by macrophages was then analyzed by flow cytometry and LSCM. As expected, both the ratio of macrophages internalized with bacteria and the amount of bacteria taken up decreased by 9% and 8%, respectively, in the presence of coating membranes (Fig. 2b, c). LSCM analysis (Fig. 2d) agreed well with the flow cytometry results. We also measured the in vivo bacterial clearance by injecting 1 × 10^7^ CFUs of CMCB into the tail vein of ICR mice. Blood was sampled and treated with lysis buffer before incubating with APC-affinity anti-CD11b-antibody for flow cytometric analysis. As shown in Supplementary Fig. 7, significantly decreased engulfment of bacteria by monocytes was observed for CMCB. Additionally, a set of PEGylated lipid-membrane-coated bacteria (PLCB) was prepared as a control (Supplementary Fig. 2e, f). As expected, PLCB also showed improved anti-phagocytosis ability comparing to uncoated EcN in vivo (Supplementary Fig. 7), indicating the decreased recognition of immune system of the host. However, the ratio of monocytes internalized with bacteria after coating with PEGylated lipid membranes increased by 46% in comparison with CMCB. The lower uptake by phagocytic cells after coating with erythrocyte membranes could be attributed to the presence of CD47 protein, a self-marker, on the surface of CMCB. These results indicate that encapsulation of EcN by cell membranes reduced in vivo bacterial clearance and therefore provided more efficient retention in blood circulation.

### In vivo inflammatory response

**Fig. 3:**
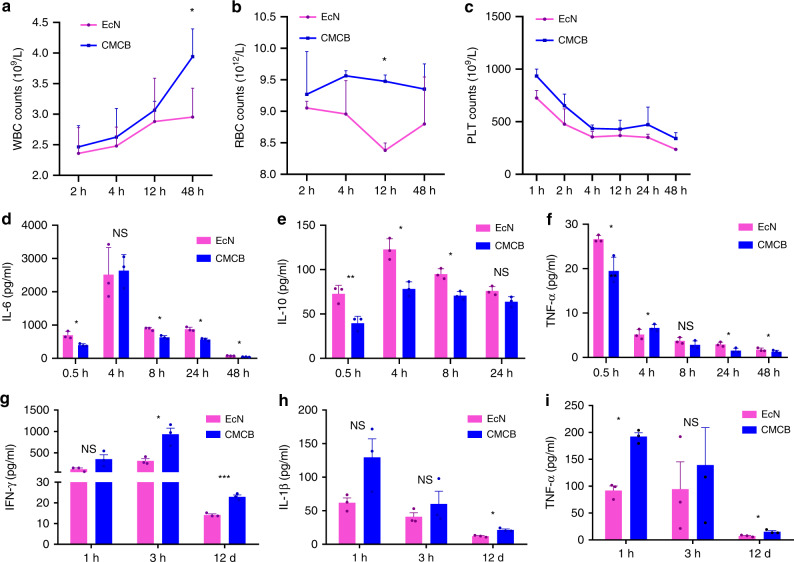
Assessment of in vivo inflammatory response. **a**–**f** EcN or CMCB (1 × 10^7^ CFUs) were injected through the tail vein and blood was withdrawn intraorbitally at the indicated time points. **a**–**c** Routine blood analysis including WBC, RBC and PLT counts. **d**–**f** Levels of cytokines in serum measured by commercially available ELISA kits, including IL-6, IL-10, and TNF-α. **g**–**i** Measurement of cytokines including **g** IFN-γ, **h** IL-1β, and **i** TNF-α in 4T1 tumor-bearing mice. Each mouse was treated with EcN or CMCB (1 × 10^7^ CFUs) through the tail vein and blood was withdrawn intraorbitally at the indicated time points. Error bars represent the standard deviation (*n* = 3). Significance was assessed using Student’s *t*-test, giving *p* values, *p < 0.05, **p < 0.01, ***p < 0.005. NS no significance

We next tested the inflammatory reaction induced by CMCB using routine blood and cytokine assays, both of which have been commonly used to evaluate the side effects caused by bacterial treatments^21^. The counts of white blood cell (WBC), red blood cell (RBC), and platelet (PLT) were quantified by a standard animal blood analyzer at predetermined time points. Compared to mice injected with uncoated EcN, mice treated with CMCB displayed higher counts of WBC^39^, RBC, and PLT (Fig. 3a–c), which are much closer to values measured for control mice injected with PBS than for mice injected with uncoated EcN (Supplementary Fig. 8). These results reveal that injection of CMCB induces a lower inflammatory response than uncoated EcN. We speculated that cytotoxins secreted from the bacteria could be encapsulated in the presence of coating membranes, which decreased the inflammatory response. More intriguingly, coating bacteria with erythrocyte membranes largely increased the detainment of the cytotoxins in contrast to PEGylated lipid membranes (Supplementary Fig. 9), which might be explained by the higher stability of the cell membranes. Cytokine assays were carried out by measuring the level of cytokines in serum sampled from mice injected with CMCB or free EcN at different time points post-injection using commercially available enzyme-linked immunosorbent assay (ELISA) kits. Similarly, uncoated EcN activated a more severe inflammatory response than CMCB. As shown in Fig. 3d–f, injection of EcN stimulated higher level of cytokines including interleukin-6 (IL-6), interleukin-10 (IL-10), and tumor necrosis factor-α (TNF-α) than the coated bacteria. Additionally, the secreted level of cytokines in mice injected with CMCB more closely approximates that of control mice treated with PBS (Supplementary Fig. 10). It is also noteworthy that the secretion of cytokines in all mice returned to its normal level in 2 weeks (Supplementary Fig. 11), verifying that embedding EcN inside a cell membrane vesicle reduced the acute side effects caused by bacteria. Tumor associated cytokines including IFN-γ, IL-1β, and TNF-α were measured by using a breast cancer 4T1 tumor-bearing mouse model. As shown in Fig. 3g-i, significant difference was observed to the levels of these cytokines at day 12 post-injection.

### Biodistribution of CMCB in tumor-bearing mice

**Fig. 4:**
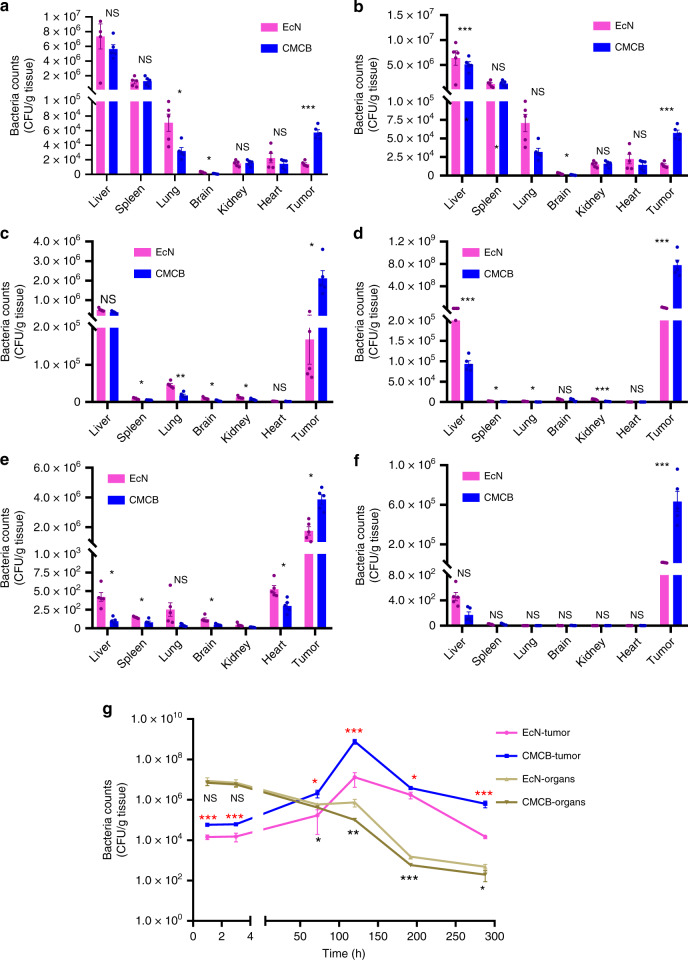
Biodistribution of bacteria in tumor-bearing mice. 4T1 tumor-bearing mice with tumor volume around 50 mm^3^ were intravenously injected with EcN or CMCB (1 × 10^7^ CFUs) and then sacrificed at indicated time points. Organ homogenates were diluted and cultured on LB agar plates at 37 °C for 24 h prior to enumeration. Biodistribution at **a** 1 h, **b** 3 h, **c** 3 days, **d** 5 days, **e** 8 days, **f** 12 days post-injection, respectively. **g** The relationship between time post-injection and bacterial number within normal organs and tumor. Error bars represent the standard deviation (*n* = 5). Significance was assessed using Student’s *t*-test, giving *p*-values, *p < 0.05, **p < 0.01, ***p < 0.005. NS no significance

Having confirmed the improved blood reservation and biocompatibility of cell membrane coated EcN, we next turned our attention to evaluate the capabilities of EcN to colonize disease sites using a 4T1 tumor-bearing nude mouse model. To avoid immune responses associated with different blood types, the cell membrane used here for biodistribution study was extracted from BALB/C mice^35^. The liver, spleen, lung, kidney, heart, brain, and tumor tissues were collected at different time points post-injection and homogenized before spreading on LB agar plates. The quantity of bacteria present in tissues was used to estimate the biodistribution. As expected, the majority of bacteria accumulated in liver and spleen following injection (Fig. 4a, b). Encouragingly, a large number of bacteria colonized tumor sites at a short time of 1 h post-injection, showing the rapid and highly efficient accumulation of EcN in tumor tissue. In particular, the bacterial number in tumor tissues from mice injected with CMCB reached up to five times higher than the mice administrated with uncoated bacteria at 1 h post-injection (Fig. 4a). The bacterial number within healthy tissues decreased significantly and continuously with the extension of time, which in turn increased dramatically in tumor tissue in the first 5 days and then remained at a relative high level for the next 7 days (Fig. 4). Remarkably, the bacterial number within tumor remained at 3240 times higher than in healthy tissues even with time increased up to 12 days post-injection of coated bacteria (Fig. 4g). The concentration of these bacteria in tumor might be ascribed to their preference for the hypoxia in the tumor tissue^21^. Furthermore, the bacterial number within tumor from mice injected with CMCB remained at 42 times higher than mice injected with uncoated bacteria 12 days post-administration. We speculate that the enhanced accumulation of bacteria in tumor tissues from mice injected with CMCB may be attributed to the high reservation in blood circulation, providing a greater opportunity to colonize tumor sites^8,18^. The colonization of EcN in liver, spleen, lung, brain, kidney, and heart from mice intravenously injected with CMCB was basically lower than that of uncoated EcN (Fig. 4a–f), which might be ascribed to the presence of an extra cell membrane that can decrease cellular uptake. These data demonstrate the superior tumor colonization ability of the coated bacteria.

### In vivo tumor imaging

**Fig. 5:**
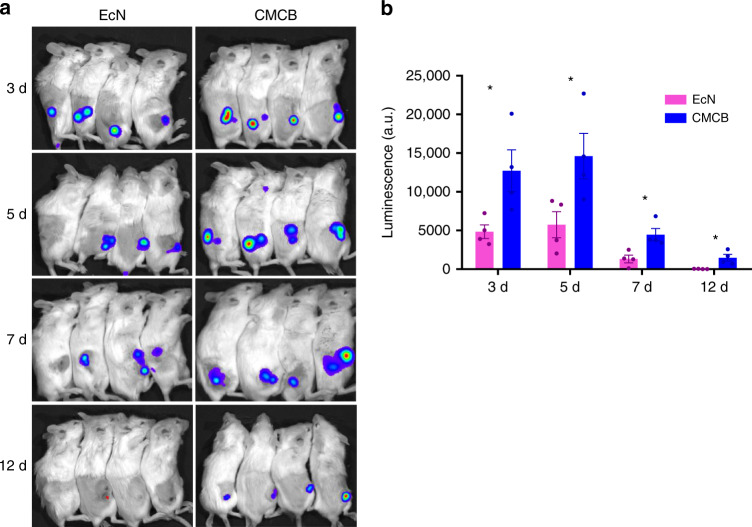
In vivo tumor imaging. **a** Tumor imaging of 4T1 tumor-bearing mice at 3, 5, 7, and 12 days post-injection of EcN or CMCB expressing LuxCDABE (1 × 10^7^ CFUs). **b** Intensity of luminescence signals from the tumor sites. Error bars represent the standard deviation (*n* = 4). Significance was assessed using Student’s *t*-test, giving *p*-values, *p < 0.05

In view of the capabilities of both fluorescence protein expression and tumor colonization specificity, we further evaluated the potential of CMCB to serve as tumor imaging agents. Conventional small molecular dyes as well as nanoprobes always suffer from short in vivo half-life and lack of targeting ability^40,41^. Here, two tumor-bearing mice models including breast cancer 4T1 and colon cancer CT26 were used for bioimaging assessments by in vivo imaging. Uncoated bacteria were utilized as a control to evaluate the imaging efficiency of CMCB. As visualized in Fig. 5a and Supplementary Fig. 12, strong luminescence signals were visualized from mice injected with luxCDABE engineered EcN. Different from conventional small molecular dyes and nanoprobes, EcN mainly appeared at the tumor sites. Notably, the average luminescence signals from the tumors of mice injected with CMCB increased to ~5 times stronger than those of mice at 3 days post-injection of EcN and almost all signals detected were concentrated at the tumor sites (Fig. 5). In addition to their excellent tumor accumulation specificity, the bacteria were able to reproduce inside the tumor tissues and maintain their luminescence signals consistently for multiple days. The signal intensity from the tumors of mice injected with CMCB remained at ~56 times stronger than that of EcN at day 12 post-injection. This unique property of CMCB could be exploited for long-term bioimaging of tumors. This data are in stark contrast with the synthetic imaging agents that their signals decreased fast as they were rapidly metabolized and eliminated^40^. The results demonstrate the potential of CMCB to serve as an efficient tool for tumor imaging.

### Removal of coating membranes

**Fig. 6:**
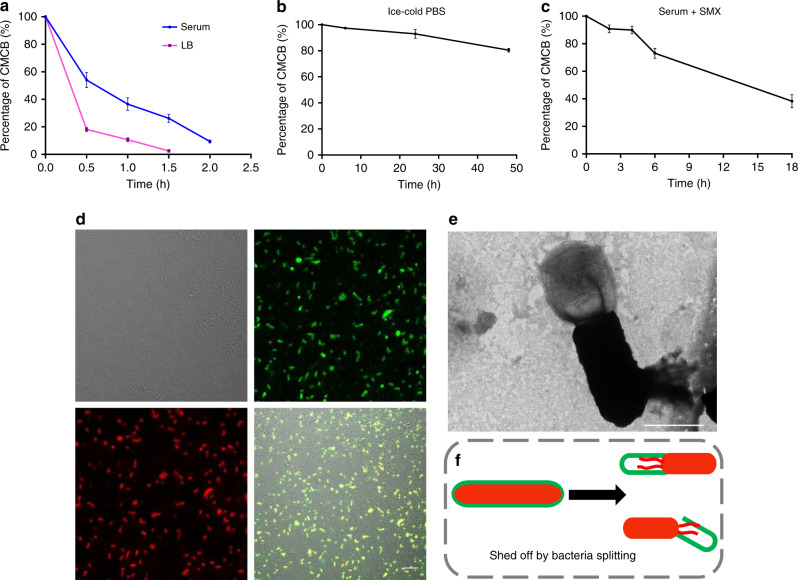
Analysis of bacterial decoating. **a**–**c** Assays for the stability of the coating membranes. The ratios of CMCB to uncoated bacteria were measured by flow cytometry at various time points after CMCB were incubated in **a** 100% serum and LB medium at 37 °C, **b** ice-cold PBS, and **c** 100% serum with 5 μg/ml of SMX. Error bars represent the standard deviation (*n* = 3). **d** Representative LSCM images of bacteria after culturing CMCB in PBS at 37 °C for 1 h. The red channel shows EcN expressing mCherry, the green channel shows cell membranes labelled with FITC-affinity anti-CD47-antibody, and the merge (light orange) shows CMCB. Scale bar, 10 μm. **e** A representative image of membrane shedding captured by TEM. Scale bar, 1 μm. **f** Schematic illustration for the removal of coating membranes

To complete our exploration of the characteristics of stealth bacteria coated with cell membranes, we finally studied the removal process of the coating membranes. Living bacteria differ from conventional surface decorated nanoparticles because they are able to proliferate even though they are surface modified with various materials^14,21,42,43^. Consequently, CMCB may shed the coating membranes during their growth and division. We examined the stability of coating membranes by incubating CMCB in different media including 100% serum, LB medium and PBS. Flow cytometric analysis shows that almost 50% and 93% of CMCB shed the cell membranes after 0.5 h and 2 h incubation in serum, respectively (Fig. 6a and Supplementary Fig. 13a, b). Interestingly, CMCB escaped from the coating cell membranes much faster when incubated in LB with only 17% remaining coated after 0.5 h incubation. We speculate the removal of coating membranes is likely attributed to the rapid division of bacteria in serum and LB. To demonstrate our hypothesis, we further tested the stability of coating membranes by incubating in ice-cold PBS and serum containing sulfamethoxazole (SMX) as the division of CMCB could be inhibited under these conditions^44,45^. As expected, we found that almost no change to CMCB after 24 h incubation in cold PBS, although the fraction of coated bacteria slightly decreased to 83% when the incubation time increased up to 48 h (Fig. 6b and Supplementary Fig. 13c). Similarly, the maintenance of coating membranes was significantly prolonged when CMCB were cultured in serum containing SMX (Fig. 6c and Supplementary Fig. 13d), verifying that the coating membranes were relatively stable and the removal of coating membranes was caused by division. The removal of coating membranes was also visualized by LSCM. As illustrated in Fig. 6d and Supplementary Fig. 14, light orange represents EcN still coated with cell membranes, while the ones in red present EcN escaped from the membrane vesicles. We also observed a large number of new-born EcN without coating membranes. This process was further captured by TEM (Fig. 6e), showing a clear uncoated EcN that was connected with an empty cell membrane vesicle. Schematic illustration for the removal of coating membranes is shown in Fig. 6f. These results suggest a controllable shedding process that the removal of coating membranes from CMCB can be easily tuned from hours to days by simply switching culture media, demonstrating CMCB are applicable for on-demand release or long-term application of the coated bacteria.

In summary, we report a strategy to engineer stealth bacteria by camouflaging with cell membranes. These camouflaged bacteria can be easily prepared by simply extruding with cell membranes. This method is applicable to diverse strains and suitable for coating with various types of cell membranes. With natural cell membrane coated, bacteria exhibit (1) a low inflammatory response that leads to fewer side effects; (2) a reduced elimination by macrophages that results in higher blood reservation; (3) low accumulation in normal organs that improves colonization abilities in disease sites; and (4) almost unchanged viabilities that remains inherent bioactivities. Giving these unique characteristics, we anticipate the broad application of this tool in diagnosis, bioimaging, and therapy applications.

## Methods

### Materials and strains

EcN, *E. faecalis*, *S. aureus*, and *Salmonella typhimurium* were purchased from China general microbiological culture collection center (GMCC, China), cell lines (CT26 and 4T1) were obtained from American Type Culture Collections (ATCC) and cultured in Dulbecco’s Modified Eagle Medium (Sigma, USA) supplemented with 10% (v/v) inactivated FBS (Sigma, USA) and 1% (v/v) antibiotic/anti-mycotic solution (Sigma, USA) at 37 °C incubator with 5% CO_2_. Regular mycoplasma evaluations were performed of the cell culture environment to ensure the absence of mycoplasma contamination. Plasmids pBBR1MCS2-Tac-mCherry, pBBR1MCS2-Tac-GFP, pMD18-luxCDABE and all other reagents were purchased from domestic suppliers and used as received.

### Growth of bacteria

EcN carrying pBBR1MCS2-Tac-mCherry, pBBR1MCS2-Tac-GFP, or pMD18-luxCDABE were grown at 37 °C overnight in LB medium with supplement of antibiotics. *E. feacialis*, *S. aureus*, and *Salmonella typhimurium* were grown at 37 °C in tryptic soy broth (TSB) medium. Overnight culture was diluted 1:50 to fresh LB medium and grown at 37 °C for 3 h. Bacteria were collected by centrifugation at 4200 × *g* for 10 min and resuspended in ice-cold PBS. Bacterial counts were determined by making dilutions of bacterial suspension, culturing them on LB agar plates at 37 °C overnight and counting the colony forming units (CFUs).

### Preparation of erythrocyte membranes

All the animal procedures complied with the guidelines of the Shanghai Medical Experimental Animal Care. Animal protocols were approved by the Institutional Animal Care and Use Committee of Shanghai Jiao Tong University School of Medicine. Red blood cell membranes were prepared in accordance with previously reported method with minor modifications^36^. Briefly, whole blood was extracted from anaesthetized male ICR mice (6–8 weeks) purchased from Jiesijie Laboratory Animal Center (Shanghai, China) through eye socket and then centrifuged at 800 × *g* for 5 min at 4 °C to remove serum and the buffy coat. After being washed in cold 1 × PBS for three times, the obtained packed RBCs were suspended in 0.25 × PBS on ice for 30 min, followed by centrifugation at 10,000 × *g* for 5 min to remove the hemoglobin. The pink pellet was washed by cold 1 × PBS and stored at −20 °C for further use.

### Preparation of CMCB

A total of 1.5 ml of bacteria sub-cultured for 3 h at 37 °C was collected, washed with ice-cold PBS twice, and resuspended in 1 ml of ice-cold PBS. The bacteria were then mixed with red blood cell membranes prepared from 1 ml of blood from a male ICR mouse or female BALB/C mouse, and extruded 11 times through a polycarbonate porous membrane (pore size: 1 μm) by using a mini extruder^35^ (Avavti Polar Lipids, USA). EcN were extruded as a control.

### Characterization of CMCB

CMCB expressing mCherry were resuspended in PBS and incubated with FITC-anti-CD47-antibody (1:5000) (561890, BD pharmingen, USA) for 30 min on ice in dark room, and washed with PBS before examination by laser scanning confocal microscopy (Leica TCS SP8, German) and flow cytometry (Beckman CytoFlex, USA).

The structure of CMCB was visualized using a transmission electron microscopy (HITACH, Japan). A drop of CMCB solution was deposited onto a carbon-coated copper grid. The sample was then washed with ddH_2_O twice, each of which lasted for 5 min. Subsequently, the sample was dried completely in air before observation.

### Preparation and characterization of PLCB

PLCB were prepared according to the reported method with minor modification^4^. Briefly, 1.5 ml of bacterial sub-culture were washed and resuspended in 1 ml of ice-cold calcium phosphate solution containing 12.5 mM of CaCl_2_. Hydrogenated soybean lecithin (Ponsure, Shanghai, China), cholesterol (Sinopharm, Beijing, China) and DSPE-PEG2000 (FITC-DSPE-PEG2000 for flow cytometry, Ponsure, Shanghai, China) were dissolved in 2 ml of chloroform at a 5.5: 4.5: 0.5 molar ratio. The resultant solution was dried at 50 °C using a rotary evaporator, resulting in a lipid film. The obtained film was hydrated in 1 ml of bacterial solution and vortexed for 30 min and then extruded 11 times through a polycarbonate porous membrane (pore size: 1 μm) by using a mini extruder^35^ (Avavti Polar Lipids, USA). The structure of PLCB was visualized using a transmission electron microscopy according to the method mentioned in section of Characterization of CMCB. The coating PEGylated lipid membranes were also examined by using FITC labeled PEG via flow cytometric analysis (Beckman CytoFlex, USA).

### Western blotting

The emptied red cell membranes, EcN and CMCB were treated with radioimmunoprecipitation assay (RIPA) lysis buffer (Beyotime, China) on ice for 15 min, and centrifuged at 4500 × *g*/min for 5 min. The supernatants were collected, mixed with loading buffer and then run on SDS-PAGE gel. The separated samples were transferred onto polyvinylidene difluoride (PVDF) membranes by semi-dry transfer using 20 volts for 60 min. The PVDF membrane was incubated in TBS/Tween with 5% skim milk at room temperature for 1 h, followed by incubating with anti-CD47 antibody (1:5000, ab192827, Abcam, USA) and secondary antibody (1:10,000, ab205718, Abcam, USA).

### Growth curves of CMCB

Bacteria were collected and washed with ice-cold PBS before mixing with pre-prepared red blood cell membranes as described in Preparation of CMCB. Both EcN and CMCB were diluted to reach an optical density (OD) value of ~0.15 in LB, and incubated at 37 °C with gently shaking. The OD value of cultures was recorded at 600 nm at various time points by nanodrop spectrophotometer (Eppendorf, German).

### Cell viability assay

Cell Viability was examined by a cell counting kit-8 (Beyotime, China). Both EcN and CMCB were diluted to an OD_600_ value of ~0.15 in LB. 180 μl of each culture medium was inoculated into a 96-well plate and cultured at 37 °C without shaking. Ten microliter of CCK-8 solution was added into each well. The OD value of cultures was recorded at 450 nm at 1 h intervals. A Multi-Detection Microplate reader (BioTek, USA) was used to measure absorbance at 450 nm.

### Bacterial competition assay

The killer bacterium EcN (Ampicillin resistant) and target bacterium STm (Kanamycin resistance, expressing mCherry) were grown at 37 °C overnight. The inhibition of CMCB, PLCB or EcN against STm was measured by microplate reader. STm was mixed with CMCB, PLCB, or EcN at a ratio of 1:100. One hundred and fifty microliters of each mixture was incubated in 96-well plate at 37 °C with shaking, the relative fluoresce units (RFUs) of mCherry expressed by STm were recorded at 580/610 nm at 1 hour interval for 12 h.

### Blood reservation study

The experiments were performed on 6–8-weeks-old male ICR mice. Three mice for each time points per group were used. To evaluate the blood reservation of CMCB, 1 × 10^7^ CFUs of bacteria were intravenously injected into mice tail veins. Fifty microliter of blood were collected through eye socket at 1, 2, 4, 12, and 48 h post-injection. Uncoated EcN were used as a control. Twenty microliters of each blood were diluted serially with PBS and 50 μl of each dilution was spread on solid LB agar plates, which were further incubated at 37 °C overnight before counting.

### Cellular uptake

*In vitro macrophage uptake*: Primary macrophage cells isolated from mouse abdominal cavity according to a reported method with minor modification^38^ were seeded at a density of 2 × 10^5^ cells per single dish in 1 ml DMEM with 10% FBS and incubated for 24 h at 37 °C. The cells were then treated with equal volume (10 μl) of EcN, PLCB or CMCB for 1 h followed by 1 h gentamycin treatment. Subsequently, after being rinsed with PBS for three times, the cells were viewed by LSCM. For quantitative analysis of cell uptake, the cells were digested and collected for flow cytometry analysis. *In vivo bacterial clearance*: One hundred microliters of blood from each mouse treated with EcN, PLCB, and CMCB as described in the section of Blood Reservation Study was withdrawn at 1.5 h post-injection and analyzed by flow cytometry. The blood samples were treated according to method reported elsewhere^46^. The red blood cells were lysed and the monocytes were incubated with APC-affinity anti-CD11b-antibody (1:1000, BD Pharmingen) at 4 °C for 1 h. The stained cells were washed with PBS and analyzed using the CytoFlex (Beckman CytoFlex, USA).

### Subcutaneous tumor models

The experiments were performed on female BALB/C mice (6–8 weeks) purchased from Shanghai Jiao Tong University School of Medicine (Shanghai, China), and bred under specific-pathogen-free (SPF) conditions for 4 days. Tumor cells (CT26 and 4T1) were collected and resuspended in PBS buffer. One hundred microliter of PBS containing 5 × 10^5^ of tumor cells was subcutaneously injected into the right flank. Mice were randomly assigned to two groups (treated with 1 × 10^7^ CFUs of EcN and CMCB expressing luxCDABE). The tumor volume of each mice was measured by caliper and estimated using formula: (width)^2^ × length × 0.5.

### Biodistribution

To study the biodistribution of EcN and CMCB, the mice bearing tumors (CT26 and 4T1) as described in section of Subcutaneous tumor models were intravenously injected with 1 × 10^7^ CFUs of EcN or CMCB. Five mice for each group were used. All the mice were imaged by in vivo imaging system (Caliper LifeSciences, USA) at indicated time points and sacrificed for analysis at predetermined time intervals. The liver, spleen, lung, heart, brain, kidney, and tumor were homogenized in a glass homogenizer. Equal weight of each homogenate was diluted serially with LB and 50 μl of each dilution was spread into LB agar plates with antibiotics before overnight incubation at 37 °C and bacteria counting.

### Routine blood test

Thirty microliters of blood from each mouse treated with EcN and CMCB as described in the section of Blood reservation study was withdrawn at predetermined time points into a 1.5 ml Eppendorf tube with EDTA, and then assayed to count the white blood cell, red blood cell and platelet using a standard animal blood analyzer (Hemavet 950FS, China).

### Cytokine assay

The mice treated as described in the section of Blood reservation study and Subcutaneous tumor models were sacrificed at predetermined time points. One milliliter of blood from each mouse was withdrawn through eye socket into a 1.5 ml Eppendorf tube without EDTA and incubated at 37 °C for 0.5 h. The serum was isolated by centrifugation at 10,000 × *g* for 5 min and then assayed for IL-6, IL-10, TNF-α, IFN-γ, and IL-1β using commercially available ELISA kits (MultiSciences Biotech, China).

### Tumor imaging

To evaluate the accumulation and imaging of EcN and CMCB in tumor, the mice bearing tumors (CT26 and 4T1) at size of ~100 mm^3^ as described in section of Subcutaneous tumor models were intravenously injected with 1 × 10^7^ CFUs of EcN or CMCB. All the mice were imaged by in vivo imaging system and sacrificed at predetermined time points.

### Reporting summary

Further information on research design is available in the Nature Research Reporting Summary linked to this article.

## Supplementary information

Supplementary Information

Reporting Summary

## Data Availability

All data presented in the paper are available from the authors upon reasonable request.
