# Author Correction: Camouflaging bacteria by wrapping with cell membranes

**DOI:** 10.1038/s41467-022-28847-y

**Published:** 2022-03-15

**Authors:** Zhenping Cao, Shanshan Cheng, Xinyue Wang, Yan Pang, Jinyao Liu

**Affiliations:** 1 Institute of Molecular Medicine, State Key Laboratory of Oncogenes and Related Genes, Shanghai Institute of Cancer, Renji Hospital, School of Medicine, Shanghai Jiao Tong University, 200011 Shanghai, China; 2 Department of Ophthalmology, Shanghai Ninth People’s Hospital, School of Medicine, Shanghai Jiao Tong University, 200011 Shanghai, China; 3 Shanghai Key Laboratory of Gynecologic Oncology, Department of Obstetrics and Gynecology, Renji Hospital, School of Medicine, Shanghai Jiao Tong University, 200011 Shanghai, China

**Keywords:** Biomaterials - cells, Cell delivery, Cancer imaging, Applied microbiology, Biomedical engineering

Correction to: *Nature Communications* 10.1038/s41467-019-11390-8, published online 06 August 2019.

This Article contains an error in Fig. 4, in which panel 4b was inadvertently copied from panel 4a. The correct version of Fig. 4 is:
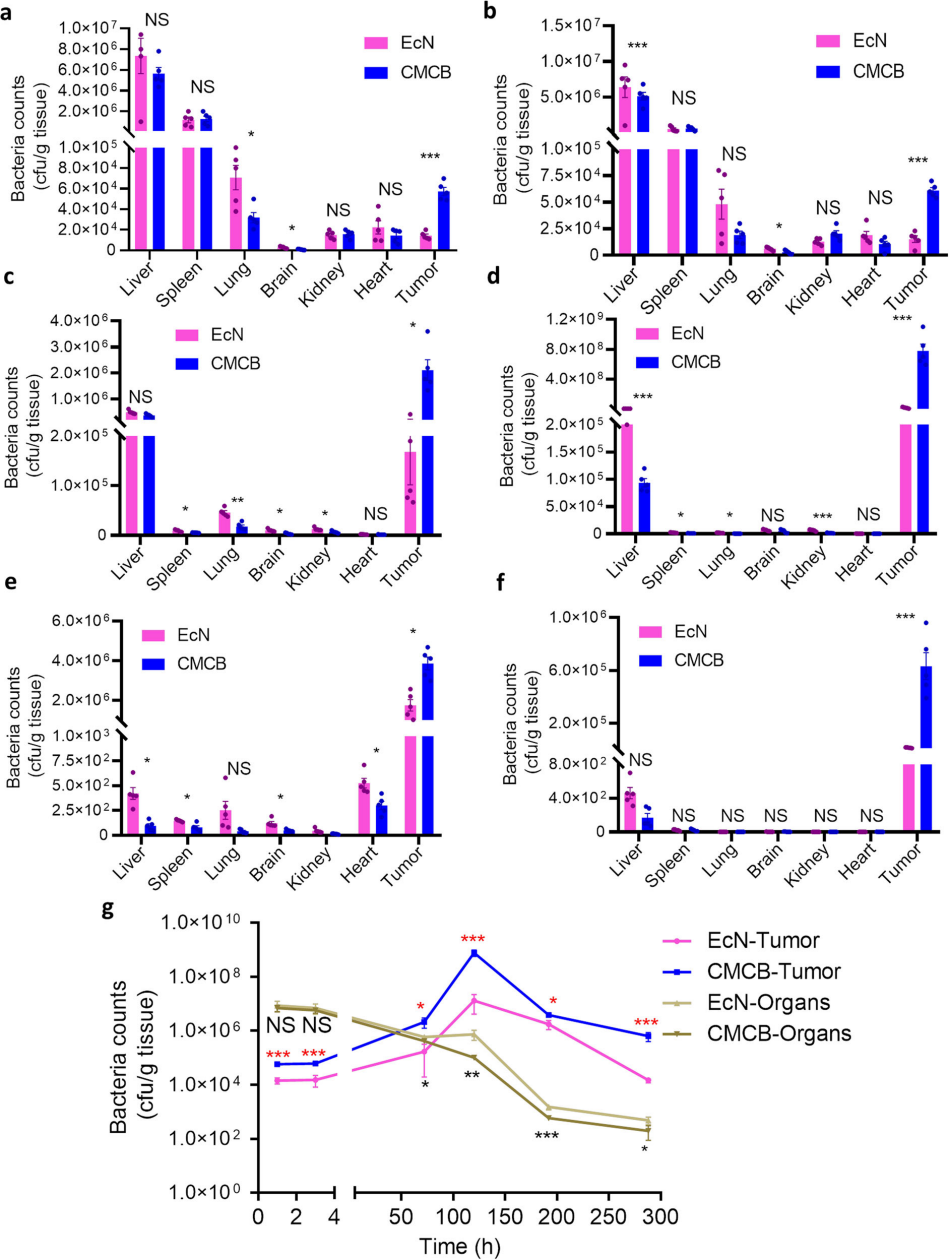


The error has not been corrected in the PDF or HTML versions of the Article.

