# Supplementary material for: Camouflaging bacteria by wrapping with cell membranes
Source: Nat Commun. 2019 Aug 6;10:3452. doi: 10.1038/s41467-019-11390-8 (PMC6684626; doi:10.1038/s41467-019-11390-8)
Supplement: Supplementary file 1 — Supplementary Information [file 41467_2019_11390_MOESM1_ESM.pdf]

Supplementary Information

Camouflaging bacteria by wrapping with cell membranes

Cao et al.

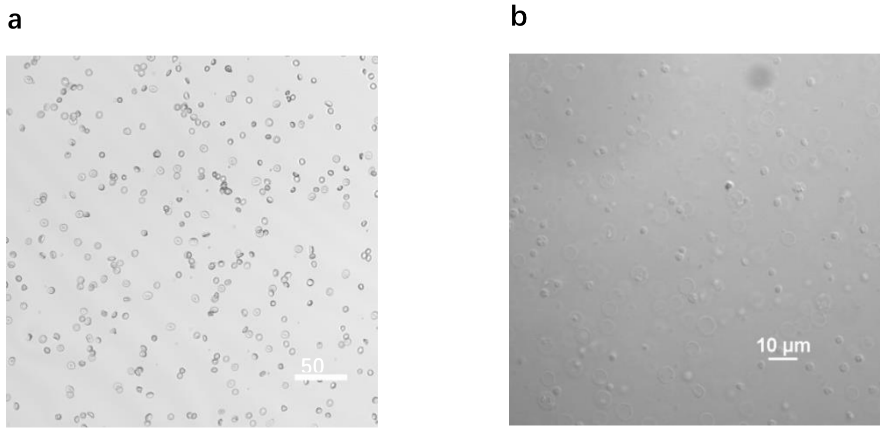

Supplementary Figure 1 Representative LSCM images of (**a**) erythrocytes and (**b**) erythrocyte membranes. The cell membranes were extracted from erythrocytes isolated from 6-8 weeks old male ICR mice.

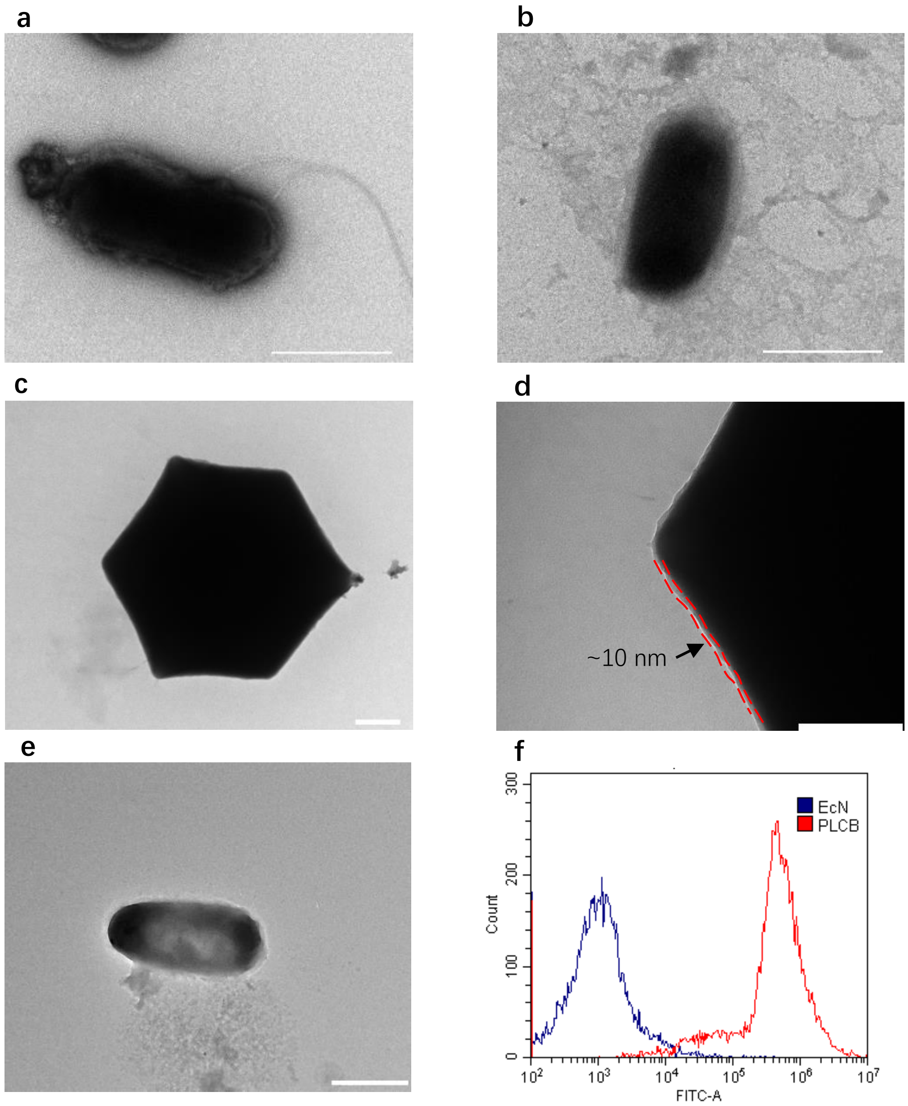

Supplementary Figure 2 Representative TEM images of (**a**) EcN and (**b**) CMCB. Scale bar, 1 μm. **c-d,** Erythrocyte cell membrane coated micro-particles. Scale bar, 200 nm. **e,** Representative TEM image of PLCB. Scale bar, 1 μm. **f,** Flow cytometric analysis of FITC-labeled PLCB.

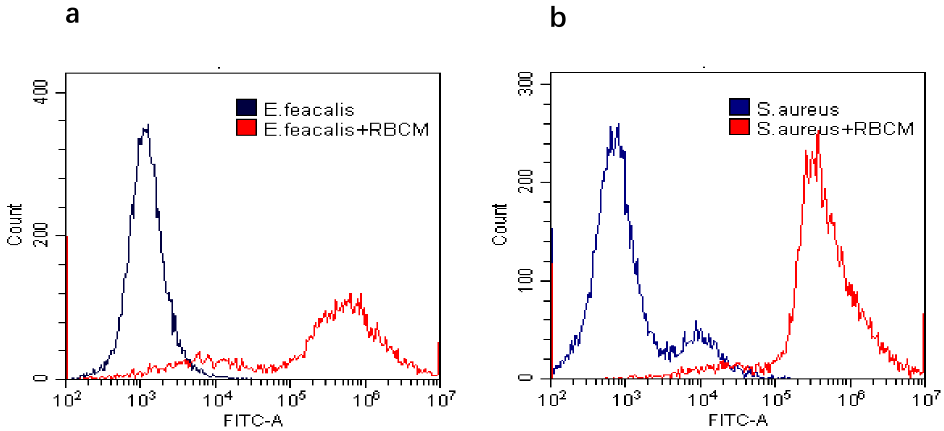

Supplementary Figure 3 Flow cytometric analysis of coated (**a**) *E. feacalis* and (**b**) *S. aureus*.

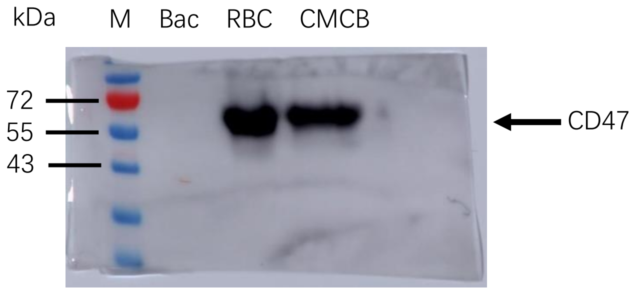

Supplementary Figure 4 CD47 protein was detected in red blood cell membranes and CMCB by western blot with anti-CD47-body (1:5000). Uncoated EcN was used as a negative control.

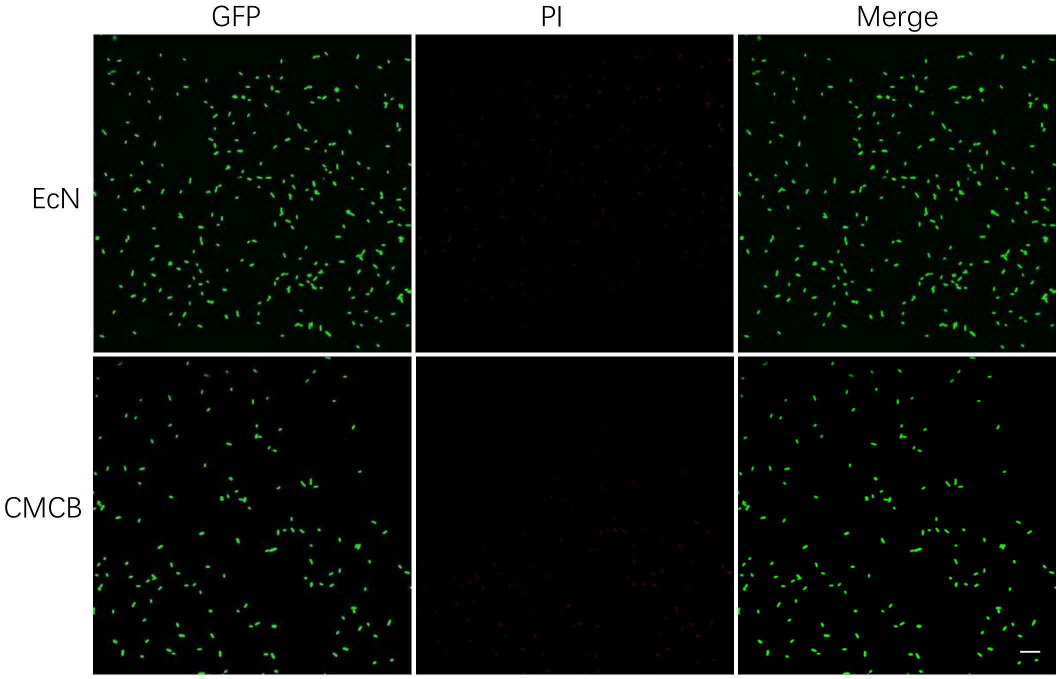

Supplementary Figure 5 Bacterial viability assay of CMCB and EcN. The green channel represents GFP and the red channel represents propidium iodide (PI). Scale bar, 10 μm.

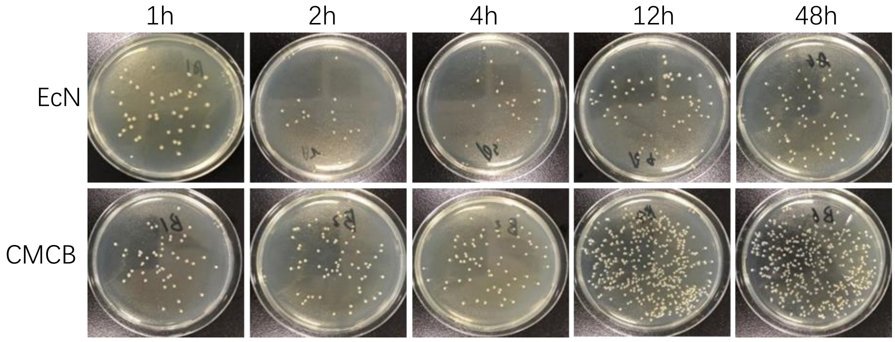

Supplementary Figure 6 In vivo blood reservation of bacteria. EcN or CMCB (1 X 10^7 CFUs) were injected through the tail vein and blood was withdrawn intraorbitally at the indicated time points, diluted to 10^{-2} and spread onto LB agar plates. Plates were incubated at 37 °C for 24 hours.

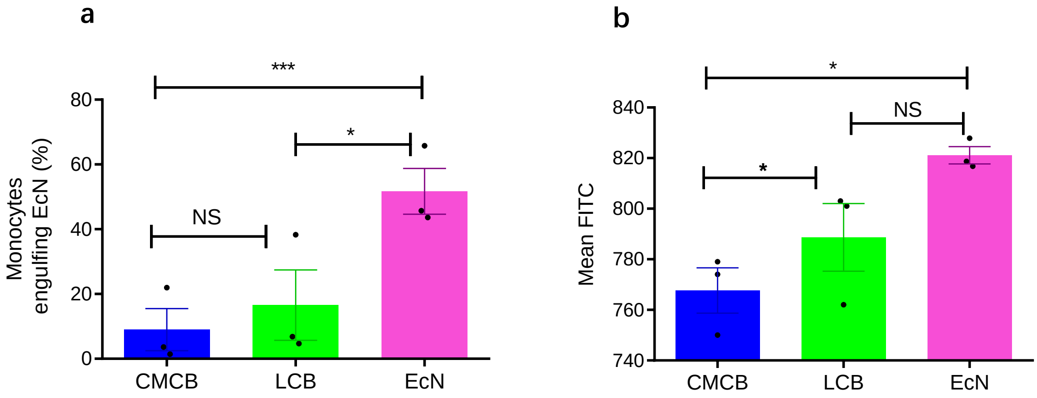

Supplementary Figure 7 Engulfment of bacteria by monocytes. **a,** The percentage of monocytes containing bacteria. **b,** the mean fluorescent intensity of the engulfed bacteria. 1 × 10^7 CFUs of EcN, PLCB or CMCB were intravenously injected into mice tail veins. 100 μl of blood were collected at 1.5 hours post-injection. The monocytes were isolated followed by red blood cells lysis, incubated with APC-affinity anti-CD11b-antibody at 4 ℃ for 1 hour and analyzed by flow cytometry. Error bars represent the standard deviation (n=3). Significance was assessed using Student's t-test, giving p values, p<0.05, *, p<0.01, **, p<0.005, ***. NS: no significance.

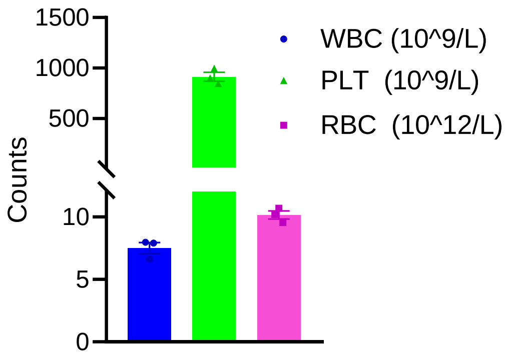

Supplementary Figure 8 Routine blood test. 100 μl of PBS was injected through the tail vein of ICR mice and 30 μl of blood was withdrawn intraorbitally at 48 hours post-injection. The counts of WBC, RBC and PLT were measured by a standard animal blood analyzer. Error bars represent the standard deviation (*n*=3).

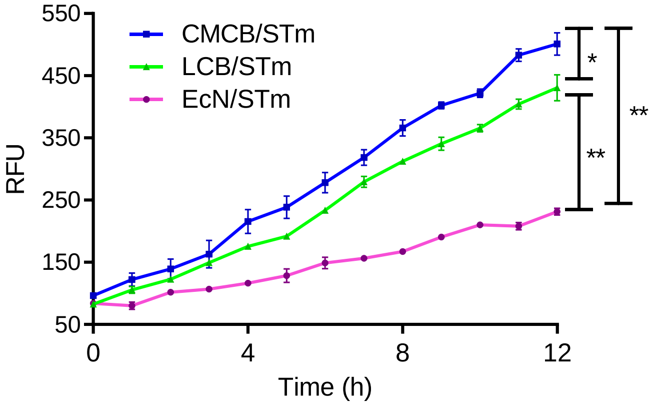

Supplementary Figure 9 Inhibition activity of EcN against STm. Equal amount of CMCB, PLCB or EcN were co-cultured with STm (expressing mCherry) at ratio of 100 : 1 in a 96-well plate at 37 ℃. The relative fluorescence units were recorded by microplate reader at 0.5 hours intervals for 12 hours. Error bars represent the standard deviation (*n*=3). Significance was assessed using Student's *t*-test, giving *p* values, $p<0.05$, *, $p<0.01$, **.

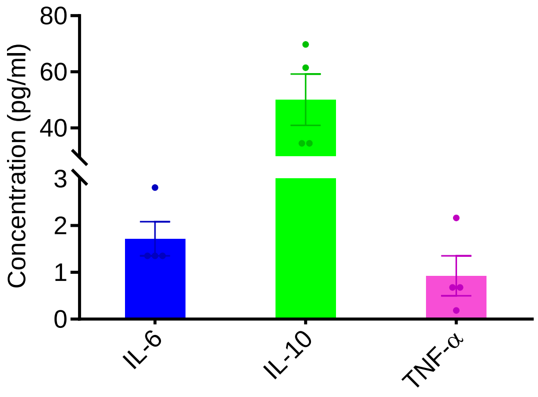

Supplementary Figure 10 Cytokine examination. Levels of cytokines (IL-6, IL-10 and TNF-α) in serums from the mice at 48 hours post-injection of PBS, which were measured by commercially available ELISA kits. Error bars represent the standard deviation (*n*=3).

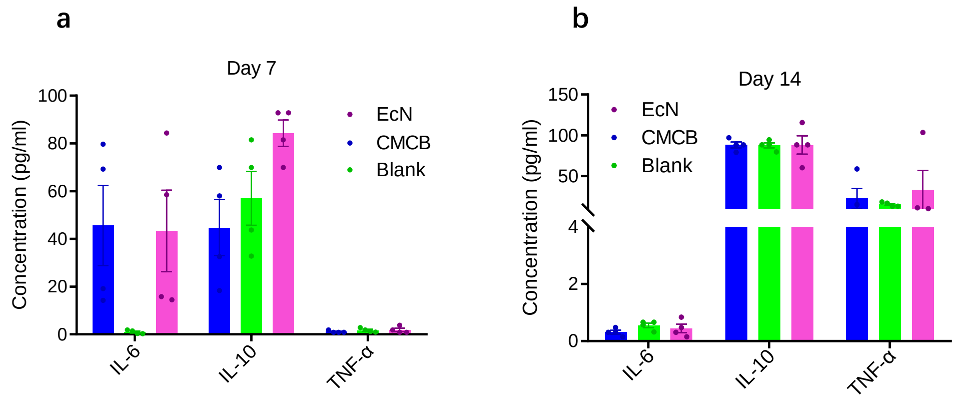

Supplementary Figure 11 Cytokine levels in serums from the mice at (**a**) 7 and (**b**) 14 days post-injection of EcN, CMCB and PBS, which were measured by commercially available ELISA kits. Error bars represent the standard deviation (*n*=3).

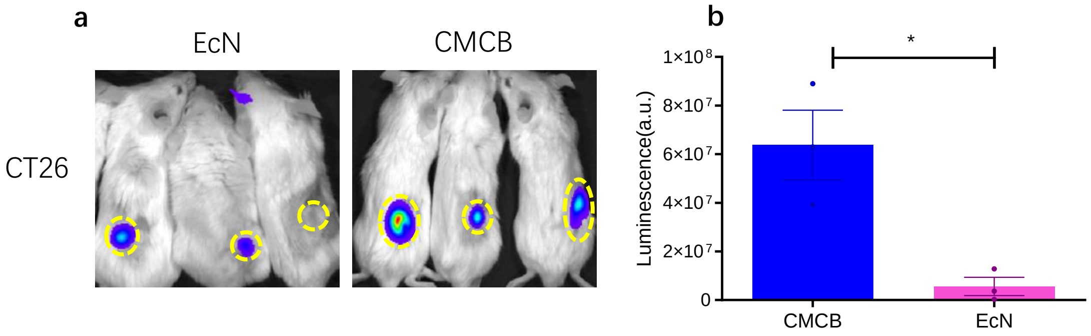

Supplementary Figure 12 In vivo tumor imaging. a, Tumor imaging of CT26 tumor-bearing mice at day 2 post-injection of EcN or CMCB expressing LuxCDABE (1 X 10^7 CFUs). **b,** Intensity of luminescence signals from the tumor sites. Error bars represent the standard deviation (*n*=3). Significance was assessed using Student's *t*-test, giving *p* values, *p*<0.05, *.

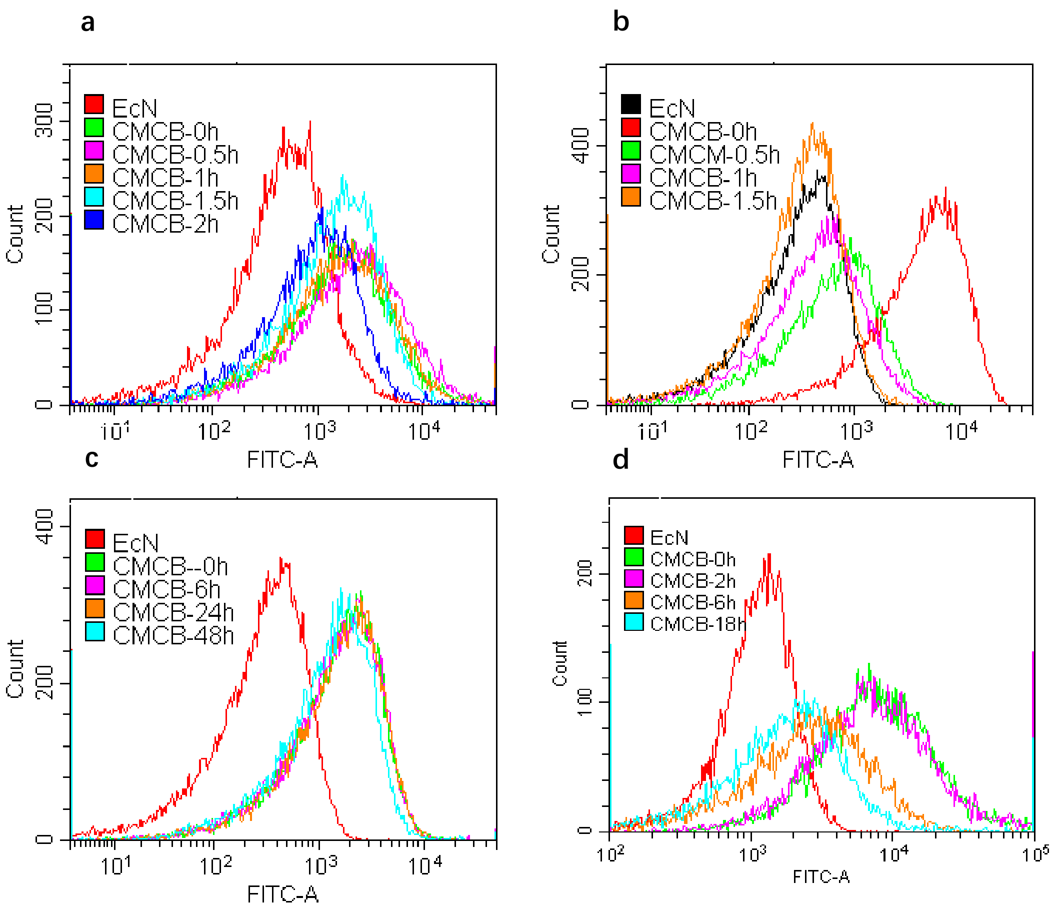

Supplementary Figure 13 Stability assay of coating membranes using flow cytometry analysis. CMCB were incubated in (**a**) 100% serum at 37 ℃, (**b**) LB medium at 37 ℃, (**c**) ice cold PBS, and (**d**) 100% serum containing 5 μg/ml of SMX. The percentages of CMCB were measured at the indicated time points.

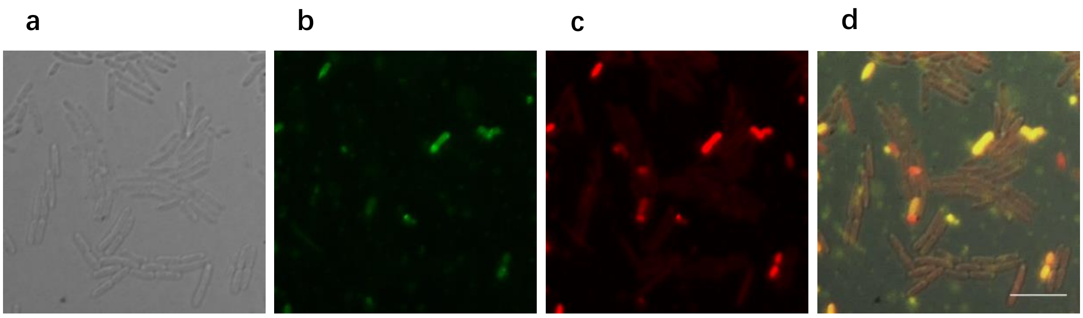

Supplementary Figure 14 Zoomed images of CMCB during the removal of coating membranes. The red channel represents EcN expressing mCherry, the green channel represents cell membranes stained with FITC-conjugated anti-CD47 antibody, and the merge (orange) represents CMCB. **a** Bright field, **b** FITC, **c** mCherry, **d** Merge. Scale bar, 10 μm.
